# Platelet hyperaggregability in high-fat fed rats: A role for intraplatelet reactive-oxygen species production

**DOI:** 10.1186/1475-2840-11-5

**Published:** 2012-01-16

**Authors:** Priscila F Monteiro, Rafael P Morganti, Maria A Delbin, Marina C Calixto, Maria E Lopes-Pires, Sisi Marcondes, Angelina Zanesco, Edson Antunes

**Affiliations:** 1 Department of Pharmacology, Faculty of Medical Sciences, University of Campinas (UNICAMP), Campinas, Sao Paulo, Brazil; 2 Department of Physical Education, Institute of Bioscience, UNESP, Rio Claro, Sao Paulo, Brazil

**Keywords:** Platelet aggregation, Obesity, Reactive-oxygen species, Nitric oxide, Cyclic GMP, BAY 41-2272

## Abstract

**Background:**

Adiposity greatly increases the risk of atherothrombotic events, a pathological condition where a chronic state of oxidative stress is reported to play a major role. This study aimed to investigate the involvement of (NO)-soluble guanylyl cyclase (sGC) signaling pathway in the platelet dysfunction from high fat-fed (HFF) rats.

**Methods:**

Male Wistar rats were fed for 10 weeks with standard chow (SCD) or high-fat diet (HFD). ADP (10 μM)- and thrombin (100 mU/ml)-induced washed platelet aggregation were evaluated. Measurement of intracellular levels of ROS levels was carried out using flow cytometry. Cyclic GMP levels were evaluated using ELISA kits.

**Results:**

High-fat fed rats exhibited significant increases in body weight, epididymal fat, fasting glucose levels and glucose intolerance compared with SCD group. Platelet aggregation induced by ADP (*n *= 8) and thrombin from HFD rats (*n *= 8) were significantly greater (*P *< 0.05) compared with SCD group. Platelet activation with ADP increased by 54% the intraplatelet ROS production in HFD group, as measured by flow cytometry (*n *= 6). N-acetylcysteine (NAC; 1 mM) and PEG-catalase (1000 U/ml) fully prevented the increased ROS production and platelet hyperaggregability in HFD group. The NO donors sodium nitroprusside (SNP; 10 μM) and SNAP (10 μM), as well as the NO-independent soluble guanylyl cyclase stimulator BAY 41-2272 (10 μM) inhibited the platelet aggregation in HFD group with lower efficacy (*P *< 0.05) compared with SCD group. The cGMP levels in response to these agents were also markedly lower in HFD group (*P *< 0.05). The prostacyclin analogue iloprost (1 μM) reduced platelet aggregation in HFD and SCD rats in a similar fashion (*n *= 4).

**Conclusions:**

Metabolic abnormalities as consequence of HFD cause platelet hyperaggregability involving enhanced intraplatelet ROS production and decreased NO bioavailability that appear to be accompanied by potential defects in the prosthetic haem group of soluble guanylyl cyclase.

## Background

Platelets play an important physiological function in haemostasis system in response to vascular injury by preventing hemorrhage [1]. Effective platelet adhesion and aggregation require the synergistic contribution of multiple receptor-ligand interactions that transmit activating signals initiating a range of platelet biochemical and morphological responses, linked to cytoskeleton remodeling, granule secretion and the generation and release of endogenous soluble agonists, such as ADP and thromboxane A_2 _(TXA_2_) [2-5].

Endothelial cell-derived nitric oxide (NO) exerts an inhibitory effect in the platelet function by activation of cGMP/PKG pathway, which in turn leads to reduction in concentration of Ca^2+^, thus preventing adhesion and aggregation of platelets to the vascular wall [6]. Nonetheless, endothelium dysfunction, present in certain pathological conditions, is characterized by a decreased NO bioavailability which incites abnormal platelet activation leading to vascular thrombosis [7,8]. Platelet dysfunction is considered an end stage of cardiovascular complications in type II diabetes mellitus, obesity and atherosclerosis that results in clinical outcomes such as myocardial infarction, stroke and peripheral artery disease [9].

Obesity is an important public health problem affecting all ages and socioeconomic groups greatly elevating the incidence of cardiovascular and endocrine-metabolic disorders. A chronic state of oxidative stress and inflammation are the hallmark of adiposity that play a pivotal role in the physiopathological events in this disorder [10,11]. These pro-inflammatory and pro-oxidant effects are associated with increased reactive-oxygen species (ROS) production and decreased NO bioavailability, which increases the risk of athero-thrombotic events [12]. Nonetheless, the exact mechanisms by which adiposity induces platelet dysfunction remain poorly investigated. In addition, most of fatal cardiovascular events as consequence of thrombotic complication are not associated with complete vascular stenosis, but rather with alterations of pro-inflammatory and pro-oxidant biomarkers, which can predict future cardiovascular events. We hypothesized that intraplatelet ROS production in adiposity contributes to thrombotic events in endocrine-metabolic disorders. Therefore, we have investigated the ex-vivo platelet reactivity in response to ADP and thrombin in high fat-fed rats, and the involvement of platelet-derived ROS and NO-cGMP pathway in modulating the platelet reactivity.

## Methods

### Animals and high-fat diet

The experimental protocols were approved by the Ethical Principles in Animal Research adopted by the Brazilian College for Animal Experimentation (COBEA) and performed in compliance with the ARRIVE guidelines on animal research [13]. Male Wistar rats were housed in temperature-controlled rooms on a 12 h light-dark cycle. The animals were housed two per cage and fed for 10 weeks with either a standard chow diet (carbohydrate: 70%; protein: 20%; fat: 10%) or a high-fat diet that induces obesity (carbohydrate: 29%; protein: 16%; fat: 55%), according to our previous work [14].

### Body Weight, epididymal fat mass and glycemia

The body weight and epididymal fat mass were evaluated in the beginning and at final time of the study. The glucose concentration was measured in blood from the tail vein (Accu-Check Performa, Roche Diagnostics, Indianapolis, IN, USA).

### Oral glucose tolerance test (OGTT) and insulin tolerance test (ITT)

Oral glucose tolerance test was performed after 12 h of fasting. Control and high-fat fed (HFF) obese rats received a 20% glucose solution (2 g/kg) by gavage. Blood samples were collected from tail vein at basal condition and after 30, 60 and 120 min of glucose loading. Whole-body insulin sensitivity was analyzed by the Insulin Tolerance Test (ITT). Venous blood samples were collected before (0 min) and 15, 30 and 60 min after an intraperitoneal injection of regular insulin (0.75 U/kg).

### Isolation of blood platelets and aggregation assays

Rats were anaesthetized with isoflurane, and blood was collected from abdominal aorta in 1:9 (v/v) of ACD-C (12.4 mM sodium citrate, 13 mM citric acid, 11 mM glucose). Platelet-rich plasma (PRP) was obtained by centrifugation of whole blood at 200 g for 15 min at room temperature. Five milliliters of PRP were added to 7 ml of washing buffer (140 mM NaCl, 0.5 mM KCl, 12 mM trisodium citrate, 10 mM glucose, 12.5 mM saccharose, pH6), and centrifuged (800 *g*, 13 min). The pellet was resuspended in washing buffer, and the procedure was repeated once. The platelets were gently suspended in Krebs solution (118 mM NaCl, 25 mM NaHCO_3_, 1.2 mM KH_2_PO_4_, 1.7 mM MgSO_4_, 5.6 mM glucose, pH 7.4). The platelet number was adjusted to 1.2 × 10^8 ^platelets/ml in the presence of 1 mM CaCl_2_. Platelet aggregation was measured in a two channel aggregometer (Chronolog Lumi-Aggregometer model 560-Ca, Havertown, PA, USA) at 37°C with stirring (1000 rpm). Platelet aggregation assays were carried using ADP (10 μM) or thrombin (100 mU/ml).

### Measurement of reactive-oxygen species (ROS) by flow cytometry

Measurement of intracellular levels of ROS was carried out according to a previous study [15]. Briefly, washed platelets (obtained as detailed above) were resuspended in Krebs-Ringer solution at 1.2 × 10^8 ^platelet/mL in the presence of 1 mM of calcium and 5 μM of 2'-7'-dichlorofluorescin diacetate (DCFH-DA). Platelet suspension (500 μL) were pre-incubated with N-acetylcysteine (NAC, 1 mM) or PEG-catalase (1000 U/ml) for 15 min before addition of DCFH-DA. Platelet suspension was then incubated or not with ADP (20 M) or H2O2 (8 mM; positive control) for 20 min. Platelet samples were then centrifuged (800 g 10 min), and the pellet was resuspended in Krebs solution (500 μL). Samples were analyzed using a Becton Dickinson flow cytometer (FACSCalibur, Becton Dickinson, San José, CA, USA) equipped with a 488 nm wavelength argon laser, 510 × 540 nm band pass filters. Platelets were identified by the forward and side scatter signals. Ten thousand platelet specific events were initially analyzed by the cytometer. Non-activated and activated platelets were gated so as not to analyze platelet aggregates and microparticles. The gates were then analyzed for mean fluorescence.

### Extraction and measurement of cGMP

Washed platelets (1.2 × 10^8 ^platelets/mL) were incubated with the phosphodiesterase inhibitor 3-isobutyl-l-methyl-xanthine (IBMX; 2 mM) for 15 min. Next, platelets were incubated with sodium nitroprusside (SNP, 10 μM), S-nitroso-N-acetylpenicillamine (SNAP, 10 μM) or BAY 41-2272 (10 μM) for 3 min, after which the reaction was interrupted by the addition of cold-acidified absolute ethanol (67%, vol/vol), and samples were vigorously agitated for 30 s. Cell samples were centrifuged (4,000 *g*, 30 min at 4°C). Supernatants were dried at 55-60°C under a stream of nitrogen. Cyclic GMP was measured by using a kit from Cayman Chemical (Ann Arbor, MI). The assays were performed in duplicates. The limit of cGMP detection is 1 pmol/mL.

### Materials

Adenosine diphosphate (ADP), thrombin, PEG-catalase, N-acetylcysteine (NAC), sodium nitroprusside, S-nitroso-N-acetylpenicillamine (SNAP) were purchased from Sigma Chem. Co. (St. Louis, MO, USA). 5-cyclopropyl-2-[1-(2-fluoro-benzyl)-1H-pyrazolo[3,4-b]pyridin-3-yl]-pyrimidin-4-ylamine (BAY 41-2272) was provided by Pharma Research Center, Bayer (Wuppertal, Germany). Iloprost was supplied by Schering (Germany).

### Statistical analysis

Data are expressed as means ± SEM of *n *rats. The statistical significance between groups was determined by using one-way ANOVA followed by the Bonferroni test. Where appropriate, unpaired Student's t test was used to compare specific groups. Significance was established at *P *< 0.05.

## Results

### Body weight, oral glucose tolerance test (OGTT) and insulin tolerant test (ITT)

**Figure 1:**
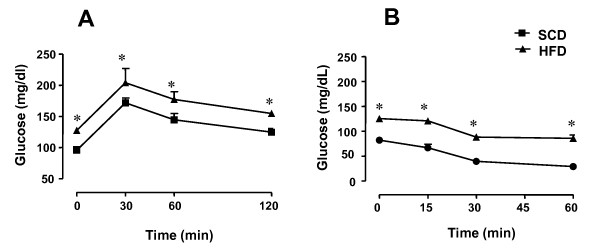
**Effect of 10-week high-fat diet (HFD) or standard chow diet (SCD) on oral glucose tolerance test (OGTT; panel A) and insulin tolerant test (ITT; panel B)**. The values represent means ± SEM for *n *= 4-6 rats each group. * p <0.05 compared with control group.

Rats treated with high-fat diet (HFD) exhibited a significant increase in body weight and epididymal fat compared with animals receiving standard-chow diet (SCD), approximately 20% and 135% (*P *< 0.05), respectively (Table 1). Fasting glucose levels were significantly higher in HFD compared with SCD group, about of 40% (*P *< 0.05). The OGTT showed that glucose levels remained increased after glucose consumption in HFD in all evaluated time-points in comparison with SCD group (Figure 1A; *n *= 4-6). Insulin sensitivity was markedly reduced in HFD compared with SCD rats (*P *< 0.05), as assessed by the ITT (Figure 1B).

**Table 1:** Effect of high-fat diet on body weight, epididymal fat weight and glucose levels

	SCD	HFD
Body weight (g)	457 ± 8.1	551 ± 17*
Epididymal fat (g)	7.6 ± 0.6	17.9 ± 1.5*
Glucose (mg/dl)	89 ± 3.8	127 ± 3.2*

Male Wistar rats were fed with either a standard chow diet (SCD) or high-fat diet (HFD) during 10 weeks.

Values represent means ± SEM animal for *n *= 4-6. *P < 0.05 compared with control group.

### ADP- and thrombin-induced washed platelet aggregation

Platelet aggregation induced by ADP (10 μM) was significantly greater (*P *< 0.05) in washed platelets obtained from HFD compared with SCD group (80.0 ± 1.1% and 59.2 ± 1.5%, respectively; *n *= 8). Similarly, platelet aggregation induced by thrombin (100 mU/ml) was significantly greater (*P *< 0.05) in washed platelets obtained from HFD compared with SCD group (81.2 ± 3.4% and 60.2 ± 3.1%, respectively; *n *= 8).

### Generation of ROS in washed platelets

**Figure 2:**
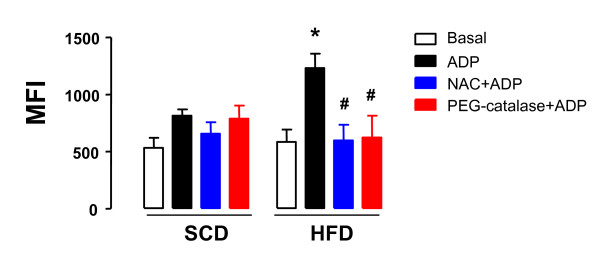
**Effect of high-fat diet in the generation of intraplatelet reactive-oxygen species (ROS)**. Male Wistar rats were fed with either a standard chow diet (SCD) or high-fat diet (HFD) during 10 weeks. Washed platelets (1.2 × 10^8 ^platelets/ml) from SCD or HFF rats were pre-incubated with N-acetylcysteine (NAC, 1 mM for 15 min) or PEG-catalase (1000 mU/ml, 15 min) and then stimulated with ADP (10 μM). Generation of ROS was quantified by flow cytometry using 2'-7'-dichloroflurescin diacetate (DCFH-DA). Results are shown as mean ± SEM values for *n *= 6-7. *P < 0.05 compared with SCD group. ^#^*P *< 0.05 compared with ADP in HFF group. MFI = mean fluorescence index.

The basal production of intraplatelet ROS did not significantly change between HFD and SCD groups (Figure 2). In SCD group, activation of platelets with ADP (10 μM) did not significantly change the ROS production compared with its respective basal ROS production. However, in HFD group a marked increase in the intraplatelet ROS production was found after activation by ADP, by about of 54% (*P *< 0.001; Figure 2). Pretreatment of platelets with NAC (1 mM) or PEG-catalase (1000 U/ml) did not significantly affect the ROS production in SCD, but prevented the increased ROS production in platelets from HFD rats (*n *= 6 each group; Figure 2).

### Effect of NAC and PEG-catalase on platelet hyperaggregability of HFF Rats

**Figure 3:**
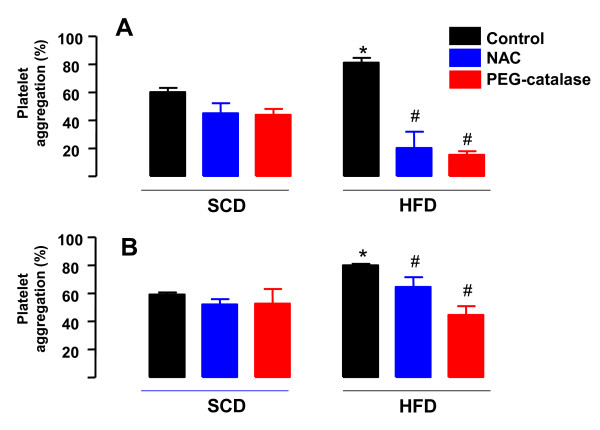
**Effect of N-Acetylcysteine (NAC) and PEG-catalase on washed platelet aggregation of rats fed with a standard chow diet (SCD) or high-fat diet (HFD) during 10 weeks**. Washed platelets (1.2 × 10^8 ^platelets/ml) from SCD or HFD rats were stimulated with thrombin (100 mU/ml; panel A) or ADP (10 μM; panel B) in the absence or the presence of NAC (1 mM) or PEG-catalase (1000 U/ml). Results are shown as mean ± SEM values (*n *= 4-7). *P < 0.05 compared with untreated control group. ^#^*P *< 0.05 compared with the respective untreated platelets.

Pretreatment of platelets with NAC (1 mM, 3 min) or PEG-catalase (1000 U/ml, 3 min) did not significantly affect thrombin- or ADP-induced platelet aggregation in SCD rats (*n *= 4-7; Figure 3). However, NAC and PEG-catalase fully prevented the platelet hyperaggregability induced by thrombin or ADP in HFD group (Figure 3).

### Effect of sodium nitroprusside (SNP), S-nitroso-N-acetylpenicillamine (SNAP) and BAY 41-2271 on platelet aggregation and cGMP intracellular levels

**Figure 4:**
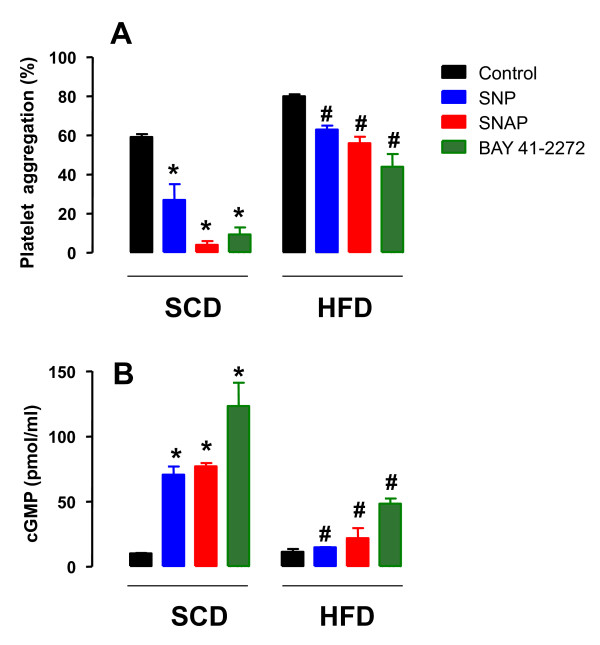
**Effect of sodium nitroprusside (SNP), S-nitroso-N-acetylpenicillamine (SNAP) and BAY 41-2272 on washed platelet aggregation (panel A) and cyclic GMP production (panel B) in rats fed with a standard chow diet (SCD) or high-fat diet (HFD) during 10 weeks**. Washed platelets (1.2 × 10^8 ^platelets/ml) from SCD or HFD rats were stimulated with ADP (10 μM) in the absence or the presence of SNP, SNAP or BAY 41-2272 (10 μM each). Results are shown as mean ± SEM values (*n *= 4-7). *P < 0.05 compared with untreated control group. ^#^*P *< 0.05 compared with the respective agent in control group.

In SCD rats, ADP-induced platelet aggregation was largely reduced by prior incubation with the NO donors SNP (10 μM; *n *= 4) and SNAP (10 μM; *n *= 5), as well as by the NO-independent soluble guanylyl cyclase stimulator BAY 41-2272 (10 μM; *n *= 7), as shown in Figure 4A. The reduction of platelet aggregation by SNP, SNAP and BAY 41-2272 were accompanied by 7.0-, 7.6- and 12.3-fold increase (*P *< 0.001) in the amounts of intracellular cGMP levels, respectively (Figure 4B). In HFD rats, inhibition of ADP-induced platelet aggregation by SNP, SNAP and BAY-412272 was markedly lower compared with SCD group (*n *= 4 each group; Figure 4A). In addition, in HFD rats, SNP and SNAP did not significantly increase the amounts of cGMP above baseline (Figure 4B). The increases in cGMP by BAY-412272 were also significantly lower in HFD compared with SCD rats (Figure 4B). Incubation of platelets with the soluble guanylyl cyclase inhibitor ODQ (10 μM, 10 min) before addition of SNP, SNAP and BAY 41-2272 (10 μM each) abolished the increase in the cGMP levels (*n *= 3; not shown).

### Effect of iloprost on platelet aggregation

Pretreatment of platelets with iloprost (1 μM, 3 min), a prostacyclin analogue that acts directly in cAMP/PKA signaling pathway, nearly abolished the ADP-induced platelet aggregation, as observed in both SCD and HFD rats (*n *= 4; Table 2). No statistical differences were found between control and HFF rats.

**Table 2:** Effect of the prostacyclin analogue iloprost (1 μM) in the ex vivo ADP-induced washed platelet aggregation

	SCD	HFD
Control	54.2 ± 2.9%	70.7 ± 4.9%^#^
Iloprost	5.5 ± 1.5%*	5.7 ± 2.2%*

Male Wistar rats were fed with either a standard chow diet (SCD, *n *= 4) or high-fat diet (HFD, *n *= 4) during 10 weeks.

*P < 0.05 compared with respective untreated platelets. ^#^*P *< 0.05 compared with untreated platelets in SCD group.

## Discussion

The present study shows that rats fed with high-fat diet (HFD) exhibit ex-vivo platelet hyperaggregability to ADP and thrombin, which is accompanied by higher intraplatelet ROS production. The platelet hyperaggregability was prevented by the antioxidant compounds PEG-catalase and NAC in HFD group indicating a critical role for intracellular ROS in this phenomenon. Moreover, the NO donors SNP and SNAP, as well as the soluble guanylyl cyclase stimulator BAY 41-2272 showed a lower efficacy in inhibiting the platelet aggregation in HFD rats, possibly as a consequence of lower platelet cGMP productions in this diet-induced obesity model.

### Platelets, Hyperglycemia and Oxidative Stress

Evidences have shown that persistent hyperglycemia can activate alternative glucose metabolism pathways, which in turn result in the formation of deleterious products derived from protein or lipid structure alterations named advanced glycation end products (AGEs), which can deeply affect the function of the cardiovascular system [16]. In vascular system, the interaction of AGES with their receptors (RAGE) can activate complex signaling pathways causing increased production of inflammatory mediators and massive ROS generation, resulting in reduced NO bioavailability and endothelium dysfunction [17], as well as alterations in coagulation system [18]. In cardiac tissues, the hyperglycemia-induced ROS activate the MEK/ERK pathway to increase GATA-4 phosphorylation, which in turn generates cardiac hypertrophy [19]. Hyperglycemia is also associated with dysregulation of sympathetic innervation to the myocardial tissues [20]. Thus, the mechanistic event by which diet-induced obesity causes platelet dysfunction may therefore be associated with hyperglycemia, which is consistent with the abnormality of the OGTT and ITT in HFD group. Previous studies show that acute hyperglycaemia enhances collagen-induced platelet aggregation via increased mitochondrial O_2_^- ^production [21]. Acute hyperglycaemia following an oral glucose tolerance test or a carbohydrate-rich meal also promotes platelet activation in vivo [22,23].

Radicals derived from oxygen represent the most important class of ROS generated in living systems. Superoxide anion (O_2_^-^) is considered the primary ROS, and it can further interact with other molecules, either directly or through enzyme- or metal-catalyzed processes, to generate other physiological relevant ROS such as hydrogen peroxyde (H_2_O_2_) and ^-^OH, as well as peroxynitrite (ONOO^-^) [24]. Adiposity in humans is reported to increase the risk of athero-thrombotic events due partly to increased oxidative stress, as evaluated by measurement of systemic biomarkers such as serum levels of lipid peroxidation, TNF-α, free fatty acids and oxidized LDL [12]. Different sources, including platelets, may generate O_2_^- ^including the NADPH-oxidase, xanthine oxidase and arachidonate-derived prostaglandin-like metabolites [25-29]. However, the contribution of intraplatelet ROS in modulating platelet reactivity in conditions of adiposity has not been explored. Therefore, this study was designed to explore the ex-vivo washed platelet aggregation in HFD rats, and the potential role of intraplatelet ROS production and NO bioavailability in modulating platelet reactivity. Our data showed that ADP- and thrombin-induced platelet aggregation were significantly higher in HFD group, which was accompanied by higher levels of ROS production, as assessed by fluorescence assays using DCFH [30]. Moreover, prior incubation of platelets with the ROS scavengers PEG-catalase or NAC suppressed both the increased ROS production and the hyperaggregability in HFD rats. Altogether, our data indicate that ex vivo platelet hyperaggregability in HFD rats is closely linked with enhanced intraplatelet ROS production. A recent study showed that NAC, at concentrations attainable with oral dosing [31], significantly reduces ADP- and thrombin-induced platelet aggregation in whole blood of type 2 diabetic patients that is associated with an enhancement of its antioxidant activity [32].

Increased oxidative stress may also influence platelet function by decreasing NO bioavailability [12]. Nitric oxide is a ROS involved in multiple biological functions essential for the cardiovascular system and platelet function. Accordingly, in our study the ADP-induced platelet aggregation was markedly reduced by the NO donors, SNP and SNAP, in SCD rats, that was accompanied by marked elevations in the cGMP levels, as expected. Interestingly, in HFD rats, platelets were resistant to the cGMP elevations in response to SNP and SNAP, as well as to the inhibitory actions of these agents on platelet aggregation. It is likely that excess of O_2_^- ^production in platelets of HFD rats inactivates SNP- and SNAP-derived NO. This is consistent with studies performed in obese subjects and type 2 diabetic obese patients where platelets are resistant to glyceryl nitrate and SNP [33,34].

### Platelet Hyperaggregability and Role of the Cyclic Nucleotides

The soluble guanylyl cyclase (sGC) is a widely distributed signal transduction enzyme that, under activation by NO, converts GTP into the second messenger cGMP which in turn affects various downstream targets such as protein kinases, cyclic nucleotide-gated channels or phosphodiesterases [35]. One of the crucial pre-requisites of the NO-mediated sGC activation is the presence of the reduced haem moiety where its oxidation or loss renders the enzyme insensitive to NO. Nitric oxide-independent sGC activators have emerged as valuable tools to elucidate the physiopathology of the NO-sGC-cGMP signaling pathway [36]. The compound BAY 41-2272 was reported as a haem-dependent and potent NO-independent sGC stimulator [37]. BAY 41-2272 directly stimulates sGC and increases the sensitivity of the enzyme to NO, generating significant amounts of cGMP by stimulating the sGC mostly via NO-independent mechanisms [38,39]. Through this mechanism, BAY 41-2272 produces a variety of effects, including anti-aggregatory effects. In our study, BAY 41-2272 greatly elevated the cGMP levels and nearly abolished the platelet aggregation in SCD rats, as expected. However, the elevations of cGMP and inhibition of platelet aggregation by BAY 41-2272 in HFD rats were significantly lower compared with SCD group. This apparently indicates that sGC of platelets from HFD rats display a defect in producing appropriate amounts of intracellular cGMP. In rat platelets, under physiological conditions, inhibition of platelet aggregation by BAY 41-2272 requires the reduced form of sGC and the presence of NO [40]. Furthermore, the free radical ONOO^- ^is able to oxidize the prosthetic haem group of sGC to its NO-insensitive Fe^3+ ^state [41-43]. If that takes place in platelets from HFD rats, then BAY 41-2272 would be indeed expected to be less effective in activating sGC. In this aspect, it would be worth trying haem-independent sGC activators such as HMR1766 and BAY 58-2667 because they prevent sGC from oxidation-induced degradation, as evidenced in chinese hamster ovary cell line and in primary porcine endothelial cells [44]. Interestingly, the direct sGC activator HMR1766 has been shown to enhance the NO/cGMP-mediated signaling in platelets from streptozotocin-induced diabetic rats, reducing platelet-aggregates with other blood cells [45].

Besides the NO - cGMP - PDE5 pathway, the activation of platelets is inhibited by cAMP-elevating agents [46]. Elevation of intracellular cAMP levels can be achieved through the activation of adenylate cyclase either directly or through appropriately coupled membrane receptors, as well as by preventing the hydrolysis of cAMP by the cyclic nucleotide phosphodiesterases. In our study, the cAMP-elevating agent iloprost (stable prostacyclin analogue) suppressed the ADP-induced platelet aggregation in both SCD and HFD groups, excluding that hyperaggregability in HFD rats reflect changes in the cAMP signaling pathway.

## Conclusions

Our findings clearly show that metabolic abnormalities as consequence of HFD in rats cause platelet hyperaggregability involving enhanced intraplatelet ROS production and decreased NO bioavailability accompanied by potential defects in the prosthetic haem group of sGC.

## List of abbreviations

ADP: adenosine 5'diphosphate; HFD: high fat diet; HFF: high fat-fed; H2O2: hydrogen peroxyde; IBMX: 3-isobutyl-l-methyl-xanthine; ITT: insulin tolerance test; NAC: N-acetylcysteine; NO: nitric oxide; OGTT: oral glucose tolerance test; ONOO-: peroxynitrite; PRP: Platelet-rich plasma; ROS: reactive-oxygen species; sGC: soluble guanylyl cyclase; SNAP: S-nitroso-N-acetylpenicillamine; O2-: superoxide anion; TXA2: thromboxane A2.

## Competing interests

The authors declare that they have no competing interests.

## Authors' contributions

AZ and EA carried out interpreting the data and writing the manuscript; PFM, RPM, MAD, MEC, MELP and SM carried out data acquisition and reviewing statistical analysis. All authors read and approved the final manuscript.
